# Medical Students’ Knowledge of Indications for Imaging Modalities and Cost Analysis of Incorrect Requests, Shiraz, Iran 2011-2012

**Published:** 2014-05

**Authors:** Parisa Islami Parkoohi, Reza Jalli, Mina Danaei, Shiva Khajavian, Mehrdad Askarian

**Affiliations:** 1 Department of Community Medicine, School of Medicine, Shiraz University of Medical Sciences, Shiraz, Iran;; 2 Shiraz Anesthesiology and Critical Care Research Center, Nemazee Hospital, Shiraz University of Medical Sciences, Shiraz, Iran;; 3 Medical Imaging Research Center, Department of Radiology, Nemazee Hospital, Shiraz University of Medical Sciences, Shiraz, Iran;; 4 Student Research Committee, Department of Community Medicine, School of Medicine, Shiraz University of Medical Sciences, Shiraz, Iran

**Keywords:** Medical students, Knowledge, Cost analysis

## Abstract

Medical imaging has a remarkable role in the practice of clinical medicine. This study intends to evaluate the knowledge of indications of five common medical imaging modalities and estimation of the imposed cost of their non-indicated requests among medical students who attend Shiraz University of Medical Sciences, Shiraz, Iran. We conducted across-sectional survey using a self-administered questionnaire to assess the knowledge of indications of a number of medical imaging modalities among 270 medical students during their externship or internship periods. Knowledge scoring was performed according to a descriptive international grade conversion (fail to excellent) using Iranian academic grading (0 to 20). In addition, we estimated the cost for incorrect selection of those modalities according to public and private tariffs in US dollars.

The participation and response rate was 200/270 (74%). The mean knowledge score was fair for all modalities. Similar scores were excellent for X-ray, acceptable for Doppler ultrasonography, and fair for ultrasonography, CT scan and MRI. The total cost for non-indicated requests of those modalities equaled $104303 (public tariff) and $205581 (private tariff).

Medical students at Shiraz University of Medical Sciences lacked favorable knowledge about indications for common medical imaging modalities. The results of this study have shown a significant cost for non-indicated requests of medical imaging. Of note, the present radiology curriculum is in need of a major revision with regards to evidence-based radiology and health economy concerns.

## Introduction

Medical imaging has a remarkable role in the practice of clinical medicine.^1^ Clinicians should not underestimate the related medical hazards of these modalities such as potential carcinogenicity of radiographies and responses to contrast solutions that range from a slight allergic reaction to intense responses such as systemic nephrogenic fibrosis.^2^^,^^3^

Currently, by taking into consideration limited resources, physicians should consider the costs before requesting imaging studies.^4^

In a study from the medical students' points of view, the capability for interpretation of diagnostic images and recognition of abnormal results showed higher priority over concerns such as indications for various medical imaging modalities, implications for using these modalities such as the adverse effects of radiographies, and costs.^5^

Researchers of a survey at Boston University found that the majority of medical students were unfamiliar with the available reference guidelines for radiologic imaging.^6^ A survey of 62 new medical graduates in New Zealand reported that students’ theoretical and practical knowledge regarding common radiological investigations was moderate. It was proposed that a structured teaching program in radiology should be offered by medical schools.^7^

Few studies have been conducted in this area in Iran. A study among 134 dentists in Yazd revealed that knowledge for the correct prescription of radiographs was not at a desired level according to the available evidence-based guideline.^8^

To our knowledge, no study has been conducted regarding medical students' awareness about indications for diagnostic imaging in Iran. In this study we assessed the level of knowledge regarding indications for five common medical imaging modalities among medical students at Shiraz University of Medical Sciences. We have estimated the imposed cost for non-indicated requests of these modalities. This survey can be a starting point for designing a qualified curriculum for radiology training courses.

## Patients and Methods

This cross-sectional survey was conducted on all medical students at Shiraz University of Medical Sciences, Southern Iran who were in their externship or internship periods. We contacted students after retrieving their names from the Medical Education Unit.

The inclusion criterion was having passed the radiology training course as identified by oral questions from the participants. Individuals who did not agree to participate were excluded. The study was conducted from September 2011 to July 2012 in university-affiliated hospitals.

Data were collected by an anonymous self-administered questionnaire designed with the collaboration of the community medicine and radiology academic staff of our research group. The questionnaire's content validity was confirmed by other academic radiology staff. Reliability of the questionnaire, as assessed by Cronbach’s alpha coefficient was 73%.

The questionnaire included 47 multiple choice questions in the form of clinical scenarios. According to an expert panel, the design and selection of the questions was based on common and important clinical conditions seen in primary health care centers that needed diagnostic radiology work ups.^9^ In the analysis step, we categorized the questions in imaging modalities according to the mentioned modality in the true item, which included ultrasonography (22 questions), CT scan (14 questions), Doppler ultrasonography (5 questions), MRI (4 questions) and X-ray (2 questions).

The questionnaires were distributed intermittently during the study period when the participants were at the hospital. In a few cases, because of participants’ duties, it was not possible to have a quick response. In those cases we requested that participants complete the questionnaire as soon as possible. The maximum time for the delayed response was one week.

Descriptive analysis of the data was done using SPSS software, version 15. We assigned a score of 1 for true responses and 0 for false or "I don't know" responses. The total and separated level of knowledge of indications for the total and for each individual imaging modality was calculated and presented according to a descriptive international grade conversion (ranging from fail to excellent) graded according to Iranian academic grading (0 to 20).^10^

Of note, at Shiraz University of Medical Sciences the only radiology course for medical students is offered during the externship period.

For cost analysis, we calculated the cost as if the knowledge resulted in the performance of an actual imaging study. 

We categorized the answers into four categories. Medical imaging modality was the top priority for diagnosis, medical imaging modality appropriate for diagnosis but it was not the top priority, no indication for any of the medical imaging modalities, and "I do not know" answers. We checked each question individually and calculated the imposed costs for each wrong answer.

In cases where participants marked no indication for any medical imaging modalities as their answer, we calculated the costs of the correct and false answers. The tariff for the no indication modality was higher or lower than the correct answer. In both situations the imposed cost was calculated as the tariff for the no indication for any modality because these modalities did not apply for diagnosis in the practice. 

In cases where participants marked the medical imaging modality that was appropriate for diagnosis however they did not recommend imaging as the top priority, if the tariff of the wrong answer was higher than the correct answer we considered the difference of the two tariffs' as the imposed cost. If the tariff of the wrong answer was lower than the correct answer, the imposed cost was calculated as zero.

For "I don't know" responses, the median of the costs of all mentioned choices was calculated as the imposed cost. 

We added all the imposed costs together and calculated the total which was subsequently divided by the total number of respondents. The result was considered to be the imposed cost per medical student. 

Finally, we converted Iranian rials into US dollars. Results were reported in US dollars according to the mean of the contemporary declared currencies reported by the Central Bank of the Islamic Republic of Iran during the study period.^11^

## Results

Of the 270 medical students invited to participate, 200 (74%) responded and completed the questionnaires. Participants included 68 female externs, 44 male externs, 52 female interns and 36 male interns.

Mean scores and categories of knowledge of indications for each modality and the total for the imaging modalities are shown in table 1. The mean knowledge score was 9.7 (fail) for all modalities. Similar scores were excellent for X-ray, acceptable for Doppler ultrasonography, and fail for ultrasonography, CT scan and MRI.

**Table 1:** Knowledge of indications for imaging modalities in medical students at Shiraz University of Medical Sciences, Iran during 2011-2012

**Modality**	**Knowledge scores**		Frequency of knowledge levels* **N (%)**				
	mean±SD^**^	Median (IQR)^***^	**Fail**	**Acceptable**	**Good**	**Very good**	**Excellent**
X-ray	16.60±5.43	20 (10-12)	7 (3.5)	54 (27)	0 (0)	0 (0)	139 (69.5)
Doppler ultrasonography	11.54±5.05	12 (8-16)	73 (36.5)	0 (0)	60 (30)	0 (0)	67 (33.5)
Ultrasonography	9.64±3.05	10 (7.27-11.86)	96 (48)	68 (34)	18 (9)	13 (6.5)	5 (2.5)
CT scan	8.61±3.55	8.57 (5.71-8.57)	116 (58)	50 (25)	15 (7.5)	15 (7.5)	4 (2)
MRI	8.05±5.05	5 (5-10)	106 (53)	50 (25)	0 (0)	38 (19)	6 (3)
Total	9.70±2.54	9.78 (8.08-11.06)	113 (56.5)	53 (26.5)	23 (11.5)	10 (5)	1 (0.5)

*Fail: 0-9.99; Acceptable: 10-11.99; Good: 12-13.99; Very good: 14-15.99; Excellent: 16-20; Range of scores: X-ray: 0-20; Doppler ultrasonography: 0-20; Ultrasonography: 1.81-19.09; CT scan: 0-17.14; MRI: 0-20; Total: 2.97-17.87; ^**^Standard deviation; ^***^Inter-quartile range

In this study, 16.5% of participants chose CT scan or MRI instead of ultrasonography for their answers. A total of 29% selected CT scan with contrast although the true answer was a scan without contrast. Approximately 14.5% of participants selected CT scan or MRI instead of X-ray modalities. 

Cost analysis for the non-indicated requests of imaging modalities is presented in table 2.

**Table 2:** Cost analysis of non-indicated requests for imaging modalities prescribed by medical students of Shiraz University of Medical Sciences, Iran during 2011-2012

**Imposed cost**	** **	**Imaging modalities**				
	**MRI**	**Radiographies**	**CT scan**	**Ultrasonography**	**Doppler ultrasonography**	**Total**
Total lost money (US$)						
Public tariff	15576.25	4575	41576.50	28567.50	14007.75	104303
Private tariff	30711.22	9038.75	81931.70	56306.48	27592.85	205581
Imposed lost money per medical student (US$^)^*						
Public tariff	77.88	23.10	208.13	142.79	70.10	522
Private tariff	153.56	45.19	409.75	281.54	137.96	1028

•Dividing total imposed costs into total number of respondents

## Discussion

Medical imaging is a support for clinical workups that can improve patient outcome.^1^ Due to the importance of economy in modern medicine, physicians should pay attention to the high cost burden of medical imaging for health care systems and communities.^4^

It is noted that in our setting, the only radiology course for medical students is presented during the externship period. Students’ knowledge during the externship period directly impacts both their practice as interns and the imposed costs.

We observed the most frequent level of total knowledge in the five common imaging modalities of X-ray, ultrasonography, Doppler ultrasonography, CT scan and MRI was fail. In another study in the United States on teaching evidence-based imaging in the radiology clerkship, it was reported that 96% of participants who were third and fourth year medical students lacked sufficient knowledge about indications and clinical effectiveness of imaging modalities.^6^

Similar to other investigators' attitude, lack of knowledge can be attributed to factors such as the lack of earlier education on radiology with regard to the basic science of radiology and the values, indications and limitations of imaging modalities and lack of appropriate guidelines.^5^ According to our close observations, other reasons could be that our medical students learn radiology concepts theoretically rather than practically in their radiology course. In addition, the attending radiologist has a passive role in students' education.

In our investigation, the majority of participants had an excellent knowledge level in terms of indications for X-ray modality. In a study in Israel, low knowledge due to inappropriate training was proposed to be one of the reasons for low perceived ability in independent interpretation of chest radiographies among third year medical students and internal medicine interns in a teaching hospital.^12^ The better results in the current study could be due to the emphasis on radiographies compared to other imaging modalities in practical radiology. Additionally, medical students encountered X-ray stereotypes more than other modalities in clinical settings.

The majority of study participants had a fail level of knowledge about indications for ultrasonography and Doppler ultrasonography. In a study about preclinical education of ultrasonography, medical students achieved a mean score of 68% for questions that pertained to clinical diagnosis with this modality in a post-training examination.^13^ There appeared to be a number of gaps in ultrasonography training such as lack of a comprehensive curriculum and no provision for making ultrasonographic imaging by medical students following clinical diagnosis.

Knowledge of indications for CT scan and MRI were not favorable in our study. It seemed that medical students were more interested in using new and sometimes more invasive diagnostic imaging methods. In an investigation about emergency department headache admissions in an acute care hospital in Singapore, 66% of the patients with headaches were prescribed either a head CT scan or MRI. Only 8% of the mentioned cases were finally diagnosed with a “potentially serious” problem according to imaging results.^14^

The calculated costs for non-indicated requests of medical imaging were high and considerable. A cost analysis of radiologic imaging in pediatric trauma patients, in a university hospital in Turkey, illustrated that the mean total cost of negative radiologic imaging per patient was $43.1.^15^

The unfavorable consequences of non-indicated requests for imaging would be more striking if we recognized that in this study, instead of the costs, it was possible to implement free health screening diagnostic tests such as mammography for breast cancer as a health concern. The numbers of equal performed public charged ($11) bilateral mammographies by the lost money in public and private tariff were $9482 and $18689, respectively. For private charged ($38), the numbers were $2745 and $5410.

In our setting, there is a significant gap in presenting health economy topics in available educational curricula for medical students, particularly those that pertain to high cost medical technologies such as imaging. This requires the collaboration of health economists in designing new curricula. 

Our study has some limitations. It would be better if the knowledge for indications of imaging modalities would be assessed just before the beginning of a radiology course as well as after completion of the course. Also, we did not consider the length of time after passing the radiology course. Because of large variations in the mentioned time, this would result in low sample sizes in the subgroups.

Researchers have introduced innovations such as provisions for making imaging by medical students, using medical students to triage off-hour diagnostic imaging requests, and involvement of radiologists through daily clinical rounds in small group discussions with medical students.^13^In the medical education context at Shiraz University of Medical Sciences, it seems that the first step would be designing a comprehensive radiology curriculum and guideline. It is suggested future investigations for medical students and radiology trainers' perspectives about the radiology training process.

## Conclusion

Medical students of Shiraz University of Medical Sciences have an unfavorable level of knowledge about indications for common medical imaging modalities. The cost of non-indicated requests of medical imaging is significant. It seems that the present radiology curriculum requires major revisions regarding evidence-based radiology and health economy concerns.
